# Associations of alcohol consumption with diabetes mellitus and impaired fasting glycemia among middle-aged and elderly Chinese

**DOI:** 10.1186/1471-2458-10-713

**Published:** 2010-11-19

**Authors:** Chen Liu, Zhijie Yu, Huaixing Li, Jing Wang, Liang Sun, Qibin Qi, Xu Lin

**Affiliations:** 1 Key Laboratory of Nutrition and Metabolism, Institute for Nutritional Sciences, Shanghai Institutes for Biological Sciences, Chinese Academy of Sciences and Graduate School of the Chinese Academy of Sciences, Shanghai, China

## Abstract

**Background:**

The U-shaped relationship between alcohol consumption and diabetes mellitus was observed among western populations. However, few studies have systematically evaluated the association in Chinese. We aimed to investigate the associations of alcohol consumption with diabetes mellitus and impaired fasting glycemia (IFG) among middle-aged and elderly Chinese.

**Methods:**

We examined 1,458 men and 1,831 women aged 50 to 70 from Beijing and Shanghai China in a cross-sectional survey. Fasting glucose, adipokines and markers of inflammation were measured. Macronutrients and alcohol consumption were assessed with standardized questionnaires.

**Results:**

Compared with abstainers, alcohol consumption was associated with a decreased risk of having diabetes mellitus in women (OR: 0.41, 95%CI: 0.22-0.78) after controlling for socio-demographic factors, physical activity, smoking, family income, family history of cardiovascular disease or diabetes, macronutrients intake, body mass index, and markers of inflammation and adipokines. In men, both low and high alcohol consumptions were associated with increased risks of having combined diabetes and IFG (ORs 1.36 [95%CI: 1.02-1.82] and 1.50 [95%CI: 1.04-2.15], respectively]. In the multivariable stratified analyses among men, moderate drinkers who had drinking days of ≥ 5 days/week had a deceased likelihood (OR: 0.61, 95%CI: 0.37-0.98) and liquor drinkers had an increased likelihood (OR: 1.47, 95%CI: 1.09-1.98) of having combined diabetes and IFG respectively, compared with the abstainers.

**Conclusions:**

An approximately J-shaped association was observed between alcohol consumption and combined diabetes and IFG among men compared with abstainers in Chinese. Whether moderate alcohol intake could help decrease diabetic risk among Chinese people warrants further investigation.

## Background

A rapid increase in the prevalence of diabetes mellitus has become one of the major public health challenges in China, with approximately 92.4 million people having diabetes [1]. Unhealthy diet and sedentary lifestyle certainly are the major determiner in diabetes epidemic. As one of the important lifestyle factors, alcohol drinking showed a U-shaped association with the risk of diabetes mellitus among western populations, with the lowest risk being observed among individuals with moderate alcohol intake [2,3]. However, contradictory findings were reported from other studies [4,5]. The inconsistency might be attributable to the differences in beverage types, drinking frequency and dietary factors. Moreover, it is uncertain whether and to what extent that genetic backgrounds as well as lifestyle factors influence the association between alcohol intake and metabolic outcomes among difference ethical populations. So far, most of studies have been conducted among western populations and only one study investigated the relationship between alcohol intake and fasting glucose in Guangzhou, a southern city of China [6]. Given the substantial variations in lifestyles and disease patterns among peoples living in different geographic locations in China, more studies based on a representative sample of Chinese people are definitely warranted.

A large body of evidence revealed that the protective effect of moderate alcohol consumption on diabetes might be explained by the improvement of insulin sensitivity [7]. Meanwhile, some studies also documented strong link of alcohol consumption with adipokines and markers of inflammation [8,9], which have been suggested to play important roles in the pathogenesis of diabetes [10,11]. However, the mediate effects of these cytokines on association between alcohol and diabetes have not been well studied in large populations.

The primary aim of this study was to investigate the associations of alcohol consumption with diabetes mellitus and impaired fasting glycemia (IFG) among middle-aged and elderly populations living in Beijing (north) and Shanghai (south) China. Furthermore, we also explored the potential modifying effects of drinking frequency, beverage type, as well as adipokines, and markers of inflammation on the association.

## Methods

### Study population

The "Nutrition and Health of Aging Population in China" study, is a population-based cross-sectional survey conducted among people aged 50 to 70 years in Beijing and Shanghai China [12]. The study was implemented simultaneously in both geographic locations from March to June 2005. A multistage sampling method was used to recruit the participants from Beijing and Shanghai. Two urban districts and one rural district in both cities were chosen and the eligible candidates listed in the residential registration record were selected randomly. All participants were provided a written informed consent and the Institutional Review Board of the Institute for Nutritional Sciences approved the study protocol. A total of 3289 participants (1,458 men and 1,831 women) were successfully recruited for the present analyses.

All subjects were interviewed in person by trained physicians or public health workers. Data of socio-demographic information, lifestyle variables and health status were collected, by using standardized questionnaires. Dietary intakes during the past year were estimated from a 74 food item Food Frequency Questionnaire (FFQ) validated by Zhao et al. [13]. Total intakes of fat, carbohydrate, protein, fiber and energy (without energy from alcohol) were calculated according to the Chinese Food Composition Table 2004 [14].

### Assessments of alcohol consumption

The information of alcohol consumption was obtained using a standardized questionnaire, which included drinking frequency, average monthly intake of each type of beverage (beer, wine, hard liquor [> 38% v/v] and light liquor [≤38%v/v]). Daily alcohol intake was calculated in grams by summing up monthly ethanol intake of each type of beverage and then divided by 30.5 with the following content: 50 ml of hard liquor, 21.85 g; 50 ml of light liquor, 15.75 g; one 640 ml-bottle of beer, 31.36 g and 50 ml of wine, 5.2 g according to the Chinese Food Composition Table 2004 [14].

Male drinkers were categorized into three groups: light (0.1-19.9 g/d), moderate (20.0-39.9 g/d) and heavy drinkers (≥40.0 g/d), and were further grouped based on varieties and frequency of alcohol consumed. Liquor drinkers were defined for those consuming more liquor than other types of alcoholic beverage, whereas non-liquor drinker were defined for those having alcoholic beverage predominately other than liquor. Due to limited female alcohol drinkers (n = 179), women were classified into abstainers and drinkers (> 0.1 g/d) in our study.

### Anthropometric measurements and laboratory methods

All study participants were invited to have a physical examination at the community clinics. Anthropometric data and fasting blood samples were collected by trained medical professional using a standardized protocol. Body weight, height, waist circumference and blood pressure were measured. Body mass index (BMI) was calculated as weight (kg)/height (m)^2^. The measurements of the plasma levels of fasting glucose, insulin, glycated haemoglobin A1c (HbA1C), high-density lipoprotein cholesterol (HDL-C), low-density lipoprotein cholesterol (LDL-C), triglyceride, C-reactive protein (CRP), interleukin-6 (IL-6), ferritin, retinol binding protein 4 (RBP4), adiponectin and plasminogen activator inhibitor type-1 (PAI-1) have been described previously [15]. HOMA-S and HOMA-B were calculated by the homeostasis model using Levy's computer model [16]. Diabetes mellitus including both type 1 and type 2 diabetes was defined as fasting glucose ≥ 7.0 mmol/L or diagnosed diabetes previously or received glucose-lowering therapy [17]. Combined diabetes and IFG was defined as fasting glucose >5.6 mmol/L or diagnosed diabetes previously or received glucose-lowering therapy.

### Statistic analysis

Forty-two individuals with acute inflammation (CRP ≥10.0 mg/L) were excluded, leaving 3,247 individuals (1,435 men and 1,812 women) for the present analyses. When using IL-6, adiponectin and PAI-1 for further analyses, another 93 individuals (36 men and 57 women) were excluded due to gross hemolysis or lipemia. Individuals who were with previously diagnosed diabetes or on glucose-lowering therapy (132 men and 143 women) were removed from the analyses for biomarkers related to glucose metabolism, while participants on oral anti-hyperlipidemia (91 men and 135 women) were excluded from the analyses for biomarkers related to lipid metabolism.

Log-transformation was used to minimize the skewness in the analyses of fasting glucose, insulin, HbA1C, HOMA-S, triglyceride, inflammatory markers, adiponectin and PAI-1. General linear model (GLM) was employed to analyze differences in the biomarkers according to the alcohol consumption categories by gender with adjustment for age, geographic location (north/south), residential region (urban/rural), educational level (≤9 or >9y in school), smoking (current smoker/former smokers/no-smokers), physical activity (low/middle/high), family income (< 20,000/20,000-39,999/≥40,000 rmb/y), family history of cardiovascular disease (CVD) or diabetes, BMI and diet (total energy intake without alcohol, energy adjusted dietary fiber and fat intake). Multinomial regression models were performed to assess the linear and nonlinear relationship between alcohol consumption and the dependent variables, using alcohol intake (g/day) as linear and quadratic terms, respectively. The multiple comparisons between different alcohol intake groups and abstainers were conducted using Dunnett's test.

The associations of alcohol consumption with the risk of having diabetes and combined diabetes and IFG were assessed by using multivariable logistic regression model with adjustment firstly for age (model 1) and then for other confounding factors (model 2). The adjustments included BMI, inflammation markers (log-transformed CRP, IL-6 and ferritin), adipokines (RBP4, log-transformed adiponectin and PAI-1) in the models 3, 4 and 5, respectively. The multivariable analyses were further stratified by drinking frequency and beverage types. All analyses were conducted using Stata 9.2 (StataCorp, college station, Texas), and *P *values <0.05 (two-side) were considered as statistical significance.

## Results

### Characteristics of study participants according to alcohol consumption

The characteristics of the study participants were summarized in Table 1. Among men, 52.1% were current drinkers and 15.3% consumed alcohol more than 40 g/d. Meanwhile, male drinkers were more likely to drink liquor and have alcohol more than 5 days per week, whereas only 9.8% female participants were current drinkers and majority of them seemed to be light, occasional and non-liquor drinkers. The proportion of drinkers was higher in Beijing (37.1% vs. 20.1%, *P *< 0.0001) and rural (51.2% vs.48.4%, *P *= 0.05) than in Shanghai and urban areas, respectively. In men, alcohol drinking was positively associated with smoking, physically activity, blood pressure, energy and dietary fat intake, and was inversely associated with family income, BMI, dietary carbohydrate and fiber intake (all *P *< 0.05). In women, drinkers tended to have higher family income and higher levels of BMI and energy intake.

**Table 1:** Characteristics of participants according to alcohol consumption categories

	Men					Women		
	
	Abstainers	0.1-19.9 g/d	20.0-39.9 g/d	≥40.0 g/d	*P *value	Abstainers	≥0.1 g/d	*P *value
Participants, n (%)	688(47.9)	386(26.9)	142(9.9)	219(15.3)		1635(90.2)	177(9.8)	
Age, y	59.4 (6.0)	58.1 (5.9)	58.1 (5.7)	58.0 (5.8)	0.60	58.5 (6.06)	58.0 (6.14)	0.28
BMI, kg/m^2^	24.0 (3.6)	24.6 (3.0)	24.0 (3.3)	23.6 (3.1)	0.0029	24.7 (3.7)	25.3 (3.9)	0.05
Waistline, cm	85.0 (11.1)	87.2 (9.3)	85.6 (10.3)	84.7 (10.1)	0.01	82.1 (10.4)	83.0 (10.2)	0.31
Systolic blood pressure, mmHg	141.2 (23.0)	142.1 (22.8)	143.2 (23.0)	146.2 (23.4)	0.04	143.6 (25.5)	140.7 (24.8)	0.15
Alcohol intake, g/d	NA	5.2 (2.1,10.3)	29.4 (23.9,31.8)	68.4 (51.6,87.7)	< 0.0001	NA	1.9(0.9,6.9)	NA
Alcohol from liquor, g/d	NA	3.0 (0.9,5.3)	15.4(11.7,19.1)	59.8 (56.8, 62.8)	< 0.0001	NA	2.6 (2.3,3.6)	NA
Alcohol from beer, g/d	NA	2.4 (0.3,4.5)	9.0 (5.6,12.4)	19.1 (16.4,21.8)	< 0.0001	NA	3.4 (2.9,3.8)	NA
Alcohol from wine, g/d	NA	1.4 (3.4)	4.4 (9.2)	6.0 (15.5)	NA	1.9 (10.0)	1.3 (0.8,1.7)	NA
Drinking frequency, %					< 0.0001			NA
< 1 day/wk	NA	40.7	4.2	0.9		NA	53.1	
1-4 day/wk	NA	29.5	21.1	9.1		NA	22.6	
5-7 day/wk	NA	29.8	74.7	90.0		NA	24.3	
Drinking pattern, %					< 0.0001			NA
Liquor drinkers	NA	43.0	56.3	75.3		NA	22.6	
Non-liquor drinkers	NA	57.0	43.7	24.7		NA	77.4	
North residence, %	38.8	66.8	56.3	55.7	< 0.0001	45.9	78.0	< 0.0001
Rural, %	51.7	40.2	52.8	67.1	< 0.0001	51.0	34.5	< 0.0001
Education >9 years, %	29.5	36.0	24.7	15.5	< 0.0001	18.4	20.9	0.41
High physical activity level, %	49.7	56.5	66.2	65.3	< 0.0001	47.0	46.3	0.67
Family income, %					< 0.0001			0.0002
< 20,000 rmb/y	30.3	21.5	26.8	38.6		27.7	16.4	
20,000-39,999 rmb/y	58.4	61.4	62.7	54.5		61.4	64.4	
≥40,000 rmb/y	11.3	17.1	10.5	6.8		10.9	19.2	
Current smoker, %	44.6	56.2	69.0	81.7	< 0.0001	4.3	15.8	< 0.0001
Family history of CVD or diabetes, %	28.8	40.4	23.2	25.1	< 0.0001	30.8	40.7	0.0075
Nutrients *								
Energy, kcal/day	2553 (855)	2622 (833)	2856 (796)	3297 (1018)	< 0.0001	2018 (566)	2212 (655)	< 0.0001
Energy without alcohol, kcal/day	2553(855)	2576(829)	2663(798)	2714(892)	0.07	2018(566)	2158(647)	< 0.0001
Fat, g/day	74.4 (25.7)	82.0 (25.9)	83.1 (27.0)	84.1(27.8)	< 0.0001	66.8 (19.2)	74.5 (17.9)	< 0.0001
Protein, g/day	81.5 (18.2)	88.0 (18.2)	87.8 (20.4)	86.4 (19.9)	< 0.0001	65.0 (14.2)	70.5 (12.7)	< 0.0001
Carbohydrate, g/day	413.2 (66.0)	392.4 (65.6)	389.7 (70.4)	389.0 (70.1)	< 0.0001	305.0 (48.2)	284.8 (42.8)	< 0.0001
Fiber, g/day	13.9 (5.8)	16.2 (6.8)	14.9 (5.3)	13.8 (5.6)	< 0.0001	12.6 (4.9)	14.8 (5.5)	< 0.0001

NA: Not applicable.

Data are means (SD) or median (inter-quartile range) where appropriate.

* All the nutrients are energy adjusted using residual regression method.

### Associations of alcohol consumption with metabolic traits and cytokines

Table 2 showed the relationships of alcohol intake with biomarkers of glucose and lipid metabolism, inflammation and adipokines. As expected, in men alcohol consumption was strongly and linearly associated with HDL and ferritin, the indicators of alcohol intake [18,19] (both *P *for linear trend ≤ 0.002). However, it was not the case among women, which might be due to the small number of female drinkers in the study.

**Table 2:** Adjusted means of metabolic traits and markers of inflammation and adipokines according to alcohol consumption categories

	Men						Women			
						
Biomarkers	Abstainers	0.1-19.9 g/d	20.0-39.9 g/d	≥40.0 g/d	*P *for linear trend *	*P *for quadratic trend†	Abstainers	> 0.1 g/d	*P *for linear trend *	*P *for quadratic trend†
Fasting glucose, mmol/l	5.49 (0.05)	5.58 (0.06)	5.47 (0.09)	5.68(0.08)§	0.10	0.93	5.45 (0.07)	5.48 (0.09)	0.40	0.66
Insulin, μU/ml	12.86 (0.34)	12.45 (0.42)	10.88 (0.52)§	11.38 (0.48)§	0.02	0.06	15.13 (0.55)	14.83 (0.71)	0.33	0.54
A1C, %	5.80 (0.04)	5.74 (0.04)	5.68 (0.06)	5.74 (0.06)	0.62	0.08	5.80 (0.05)	5.76 (0.07)	0.59	0.46
HOMA-S, %	67.9 (1.8)	70.2 (2.4)	80.5 (3.9)‡	76.3 (3.2)§	0.27	0.05	58.5 (2.1)	59.3 (2.8)	0.41	0.62
HOMA-B, %	111.9 (2.3)	103.7 (2.9)§	99.5 (4.1)§	97.3 (3.6)‡	0.008	0.14	124.5 (3.5)	121.8 (4.6)	0.16	0.37
HDL, mmol/l	1.17 (0.02)	1.20(0.02)	1.30(0.03)‡	1.36(0.02)‡	< 0.0001	0.03	1.30 (0.02)	1.33 (0.03)	0.99	0.88
LDL, mmol/l	3.12 (0.04)	3.05 (0.05)	3.05 (0.08)	2.92 (0.07)§	0.02	0.17	3.24 (0.07)	3.30 (0.10)	0.17	0.49
Triglyceride, mmol/l	1.15 (0.03)	1.17 (0.04)	1.09 (0.06)	1.05 (0.05)	0.22	0.64	1.26 (0.05)	1.18 (0.07)	0.77	0.65
CRP, mg/l	0.76 (0.04)	0.71 (0.05)	0.70 (0.07)	0.66 (0.06)	0.55	0.80	0.69 (0.05)	0.61 (0.06)	0.17	0.14
IL-6, pg/ml	1.09 (0.04)	0.97 (0.05)	1.16 (0.08)	1.05 (0.07)	0.43	0.50	1.02 (0.06)	0.95 (0.07)	0.46	0.58
Ferritin, μg/ml	152.5 (4.6)	156.7 (5.9)	165.9 (9.3)	170.9 (8.3)§	0.002	0.25	106.9 (4.5)	107.5 (6.1)	0.53	0.61
RBP4, ng/ml	40.4 (0.6)	44.1 (0.7)‡	44.4 (1.1)‡	46.4 (0.9)‡	< 0.0001	< 0.0001	39.0 (0.8)	41.5 (1.1)‡	0.005	0.01
Adiponectin, μg/ml	12.2 (0.5)	12.4 (0.6)	12.4 (0.9)	11.5 (0.7)	0.28	0.63	13.2 (0.7)	13.7 (1.0)	0.48	0.66
PAI-1, ng/ml	2.73 (0.42)	4.20 (0.81)	1.77 (0.51)	4.67 (1.16)	0.32	0.54	5.39 (1.16)	6.26 (1.86)	0.17	0.06

Data are means (SE) or geometric means (SE) adjusted for age, geographic location (north/south), residential region (urban/rural), educational level, smoking, physical activity, family income, family history of CVD or diabetes, BMI and diet (total energy intake without alcohol, energy adjusted dietary fiber and fat intake). Participants who were previously diagnosed diabetes or on glucose-lowering therapy (men: n = 132; women: n = 143) were excluded from the analyses for diabetes-associated traits, and those who were on oral anti-hyperlipidemia (men: n = 91; women: n = 135) were excluded from the analyses for lipid profiles.

* *P *values are from analyses the model alcohol consumption (g/d) as linear terms.

† *P *values are from analyses the model alcohol consumption (g/d) as quadratic terms.

‡ *P *< 0.001 compared with abstainers

§*P *< 0.05 compared with abstainers

In the multivariable analyses among men, alcohol consumption was associated with increased RBP4 and ferritin, decreased insulin, HOMA-B and LDL in linear relations (All *P *for linear trend ≤ 0.02), and associated with insulin sensitivity in quadratic relation (*P *for quadratic trend = 0.05). Compared with non-drinkers, heavy drinkers have higher levels of glucose (5.68 vs. 5.49 mmol/l, P < 0.05). In women, a positively linear association was observed between alcohol and RBP4 (*P *for linear trend = 0.005).

### Associations of alcohol consumption with diabetes and IFG

In general, alcohol consumption was not significantly associated with the risk of having diabetes in men. However, those of heavy and light male drinkers showed significantly higher risks of having combined diabetes and IFG (ORs 1.58 [95%CI: 1.12-2.22] and 1.40 [95%CI: 1.07-1.85], respectively), after adjustment for socio-demographic factors and lifestyles. In women, alcohol consumers showed a decreased risk (OR: 0.39, 95% CI: 0.21-0.73) of having diabetes compared with abstainers (Table 3, model 2).

**Table 3:** Odds ratios (95% confidence intervals) for diabetes and combined diabetes and IFG according to alcohol consumption categories

		**Men**				**Women**	
		
		**Abstainers**	**0.1-19.9 g/d**	**20.0-39.9 g/d**	**≥40.0 g/d**	**Abstainers**	**≥0.1 g/d**
		
Diabetes	cases/non-cases, n	95/593	74/312	13/129	32/187	206/1429	12/165
	Model 1	1.0 (reference)	1.53 (1.09,2.14)	0.65 (0.35,1.20)	1.11 (0.72,1.71)	1.0 (reference)	0.51 (0.28,0.94)
	Model 2	1.0 (reference)	1.25 (0.87,1.79)	0.72 (0.38,1.37)	1.57 (0.97,2.54)	1.0 (reference)	0.39 (0.21,0.73)
	Model 3	1.0 (reference)	1.26 (0.88,1.81)	0.74 (0.39,1.40)	1.61 (0.99,2.60)	1.0 (reference)	0.39 (0.21,0.73)
	Model 4	1.0 (reference)	1.30 (0.90,1.89)	0.69(0.36,1.32)	1.56 (0.96,2.53)	1.0 (reference)	0.42 (0.22,0.78)
	Model 5	1.0 (reference)	1.27 (0.87,1.86)	0.70 (0.37,1.35)	1.59 (0.96,2.61)	1.0 (reference)	0.41 (0.22,0.78)
Combined diabetes and IFG	cases/non-cases, n	270/418	211/175	52/90	107/112	596/1039	75/102
	Model 1	1.0 (reference)	1.86 (1.44,2.39)	0.89 (0.61,1.29)	1.47 (1.08,2.00)	1.0 (reference)	1.31 (0.96,1.80)
	Model 2	1.0 (reference)	1.40 (1.07,1.85)	0.81 (0.54,1.20)	1.58 (1.12,2.22)	1.0 (reference)	0.97 (0.69,1.36)
	Model 3	1.0 (reference)	1.42 (1.07,1.87)	0.82 (0.55,1.23)	1.64 (1.16,2.33)	1.0 (reference)	0.98 (0.70,1.38)
	Model 4	1.0 (reference)	1.38 (1.03,1.84)	0.77 (0.51,1.16)	1.47 (1.03,2.10)	1.0 (reference)	1.03 (0.72,1.47)
	Model 5	1.0 (reference)	1.36 (1.02,1.82)	0.80 (0.53,1.22)	1.50 (1.04,2.15)	1.0 (reference)	1.03 (0.72,1.47)

Model 1: Adjusted for age

Model 2: Adjusted for age, geographic location (north/south), residential region (urban/rural), educational level, smoking, physical activity, family income, family history of CVD or diabetes, and diet (total energy intake without alcohol, energy adjusted dietary fiber and fat intake).

Model 3: Adjusted variables in model 2 plus BMI

Model 4: Adjusted variables in model 3 plus inflammatory markers (log-transformed CRP, IL-6 and ferritin)

Model 5: Adjusted variables in model 4 plus adipokines (RBP4, log-transformed adiponectin and PAI-1)

Further adjustment for BMI, inflammatory factors and adipokines did not appropriately alter the association between alcohol drinking and the reduced risk of having diabetes in both sexes. For men, the associations of low and high alcohol consumption with the risk of having combined diabetes and IFG were only slightly attenuated but still remained statistically significant (ORs 1.36 [95%CI: 1.02-1.82] and 1.50 [95%CI: 1.04-2.15], respectively).

When using different alcohol consumption categories as reference group, moderate alcohol consumption was found to be associated with a decreased risk of having diabetes and/or IFG compared with both of heavy (ORs 0.44[95%CI: 0.22-0.91] for diabetes and 0.53 [95%CI: 0.33-0.86)] for combined diabetes and IFG) and light alcohol consumption (OR 0.59 [95%CI: 0.38-0.91] for combined diabetes and IFG) (Additional file 1).

### Associations of drinking frequency and beverage type with diabetes and IFG

In men, the associations of alcohol intake with diabetes and combined diabetes and IFG were analyzed based upon the categorized beverage type and drinking frequency. Those who drunk more than 5 day per week were further stratified according to the levels of alcohol intake (Table 4). A decreased risk (OR: 0.61, 95%CI: 0.37-0.98) of having combined diabetes and IFG was observed among moderate drinkers compared with abstainers after controlling for all of the covariates, whereas an increased risk was observed among light and heavy drinkers. In the stratified analyses according to beverage types, liquor drinkers had a higher likelihood of having combined diabetes and IFG in the full model analysis (OR: 1.47, 95%CI: 1.09-1.98), and the increased risk was mainly observed in light and heavy liquor drinkers compared with abstainers. There was no association observed among non-liquor drinkers.

**Table 4:** Odds ratios (95% confidence intervals) for diabetes and combined diabetes and IFG in men according to the categories of drinking frequency and types of beverage

		Participants (n)	Diabetes	Combined diabetes and IFG
Drinking frequency				
Abstainers		688	1.0 (reference)	1.0 (reference)
< 1 day/wk		165	1.17 (0.71,1.92)	1.45 (0.99,2.13)
1-4 day/wk		164	1.22 (0.73,2.05)	1.27 (0.86,1.88)
5-7 day/wk	0.1-19.9 g/d	115	1.24 (0.70,2.21)	1.59 (1.02,2.48)
	20-39.9 g/d	106	0.72 (0.36,1.46)	0.61 (0.37,0.98)
	≥ 40.0 g/d	197	1.72 (1.03,2.87)	1.50 (1.02,2.18)
Beverage type				
Abstainers		688	1.0 (reference)	1.0 (reference)
Liquor drinkers				
	0.1-19.9 g/d	166	1.60 (0.99,2.61)	1.85 (1.24,2.77)
	20-39.9 g/d	80	0.95 (0.44,2.03)	0.92 (0.55,1.56)
	≥ 40.0 g/d	165	1.52 (0.86,2.68)	1.49 (1.00,2.23)
	Total	411	1.43 (0.96,2.12)	1.47 (1.09,1.98)
Non-liquor drinkers				
	0.1-19.9 g/d	220	1.07 (0.68,1.70)	1.11 (0.78,1.56)
	20-39.9 g/d	62	0.40 (0.12,1.34)	0.68 (0.36,1.25)
	≥ 40.0 g/d	54	1.92 (0.87,4.23)	1.64 (0.88, 3.03)
	Total	336	1.05 (0.70,1.58)	1.08 (0.80,1.46)

Data were adjusted for age, geographic location (Beijing/Shanghai), residential region (urban/rural), educational level, smoking, physical activity, family income, family history of CVD or diabetes, diet (total energy intake without alcohol, energy adjusted dietary fiber and fat intake), BMI, inflammatory markers (log-transformed CRP, IL-6 and ferritin), adipokines (RBP4, log-transformed adiponectin and PAI-1).

## Discussion

In the present study, among men both high and low alcohol consumptions were associated with a higher risk, whereas moderate alcohol consumption with weekly drinking frequency over 5 days was associated with a lower risk of having combined diabetes and IFG. The associations were independent of lifestyle factor, inflammatory markers and adipokines. Among women, alcohol consumption was inversely associated with the risk of diabetes. To our knowledge, this is the first study investigating the associations of alcohol consumption with risks of diabetes mellitus and combined diabetes and IFG with simultaneously considering the modifying effects of drinking frequency, beverage types, as well as adipokines and markers of inflammation among Chinese people.

Previously, protective effects of moderate drinking on the development of diabetes have been observed in a large number of epidemiological studies [4,20-22]. For instance, a meta-analysis including 13 cohorts indicated moderate alcohol intake decreased the risk for diabetes by 28% [2]. However, unlike findings from previous studies, the favorable effect of moderate drinking was only evidenced when compared with heavy drinkers but not with abstainers in our study. Additionally, among men we found a reduced risk for combined diabetes and IFG in moderate drinkers with the highest level of drinking frequency. Due to the small sample size in the subgroup, it remains to be elucidated whether moderate alcohol consumption with lower drinking frequency also has favorable impact. Among women, alcohol intake was associated with a decreased risk of having diabetes mellitus but not with combined diabetes and IFG. This disparity might be explained by the limited sample size and unclear drinking pattern.

In the majority of prospective studies, high alcohol consumption increased the risk of type 2 diabetes compared to moderate drinkers. However, the association was inconsistent when compared to abstainers [2]. In this study, we observed an adverse effect among heavy drinkers compared with both moderate drinkers and abstainers. Similar effects were also found among light drinkers in disagreement with previous studies [2,3]. In previous studies, the benefit effects of alcohol on diabetes were more apparent among those who consumed wine and beer, whereas liquor drinking were suggested to have unfavorable effect [23,24]. In line with these studies, we observed that an increased risk of combined diabetes and IFG was associated with light and heavy liquor drinking but not with non-liquor drinking. Given the fact of much higher proportion of liquor drinkers in Chinese than in Caucasian [25], the controversial results between our study and others might be attributable to the differences in the types of beverage consumption. Moreover, drinking frequency might also modify the association between alcohol consumption and diabetes. In the stratified analyses, individuals drinking less than 1 d/wk tended to have a higher risk of combined diabetes and IFG. Although the majority subjects in this group were light and non-liquor drinkers, we could not rule out the possibility that some of them consumed excessive alcohol over a short period of time. Interestingly, inconsistent with previous reports [23], the increased risk was also observed in regular light drinkers (5-7 d/wk, 0.1-19.9 g/d). Given the cross-sectional design, it is unclear whether some of the individuals have recently changed drinking habit according to their health conditions. Certainly, prospective studies are needed to address this issue.

It has been suggested that the association between alcohol consumption and disease such as type 2 diabetes might be attributed to other lifestyle factors [26]. However, a large prospective study indicated that the associations do not seem to be confounded by other lifestyle behaviors [27]. Interestingly, in our study, male heavy drinkers appeared to have the lowest socioeconomic status (SES) indicated by lower educational attainment and family income. They were also more likely to have unhealthy lifestyles such as cigarette smoking, consuming more dietary fat and less fiber intake. However, controlling these confounding factors did not largely attenuate the association between alcohol consumption and combined diabetes and IFG, suggesting that effect of alcohol intake was independent of other lifestyle factors.

In line with previous studies [9,28], we found a higher level of insulin sensitivity index among male drinker compared to abstainers, reaching its peak in moderate alcohol intake, and also observed an inverse association between alcohol intake and beta-cell function after multiple adjustment, although no such effects were detected among female drinkers who were mostly light drinkers. Mechanistic linking between the levels of alcohol intake and pathogeneses of diabetes mellitus are rather complicated and has not been fully elucidated. Nonetheless, existing data from observational [29,30] and/or intervention studies [7,9] suggested that moderate drinking was associated with increased insulin sensitivity, whereas high alcohol intake showed no or even opposite effects. Studies in vitro and in vivo also indicated toxic effects of alcohol on pancreatic islet cells as well as inhibition of insulin secretion [31,32]. Recently, Crandall JP *et al*. reported that alcohol consumption was associated with lower insulin secretion based upon the results of a long-term interventional trial [28]. Taken together, the U-shaped relationship between the levels of alcohol intake and the risk of type 2 diabetes might be explained by altered insulin sensitivity and impaired beta-cell function.

Obesity, chronic inflammation and certain adipokines have been proposed to be the mediators for the association between alcohol intake and diabetes [33]. However, we only observed a minor alteration in the associations between alcohol intake and risks of having diabetes or IFG following adjustment for BMI, adipokines and markers of inflammation, implicating that these factors might not be essential players, at least in our study population. Meanwhile, one of the novel findings in our study is the strong positive linear association between plasma RBP4 concentration and alcohol intake in both sexes. Indeed, Fernandez-Real JM *et al*. recently found a positive correlation between serum RBP4 and ferritin from a cross-sectional study. In addition, they also reported that iron depletion intervention reduced serum RBP4 concentration in diabetic patients [34]. Considering the close association between alcohol consumption and elevated ferritin in our and other studies [19], the positive correlation between RBP4 and alcohol consumption might be, at least partially, explained by raised iron storage.

There were some limitations in our study. Firstly, alcohol consumption and drinking frequency were evaluated by questionnaire, which might induce recall bias. Nevertheless, the dose-response relationships between alcohol consumption and alcohol related biomarkers such as HDL and ferritin, suggested the validity of our questionnaire for the assessment of alcohol intake. Secondly, the cross-sectional nature of the current study could not allow us to establish any causal relationship. Thirdly, due to lacking of relevant data, we could not exclude former drinkers from abstainers, which might confound the observed associations between alcohol consumption and risk of having altered glucose metabolism. However, according to the data from a large cohort study conducted in 66,743 Chinese men in Shanghai [35], only 5.3% of abstainers were former drinkers. Likewise, the biases driven by misclassifying former drinkers in abstainers might be negligible. Fourthly, we could not distinguish individuals with type 1 diabetes from those with type 2 diabetes. Given that in the present study only 14.4% of diabetic individuals had a diagnosis when they were younger than 50 years, and the fraction of type 1 diabetes is less than 10% among diabetic patients, it is less likely that the associations of alcohol consumption and diabetic risk could be confounded with the autoimmune diabetes. Obviously, further prospective studies with larger sample size are needed to establish casual role of alcohol intake in pathogenesis of diabetes mellitus.

## Conclusions

In our study, an approximate J-shaped association was observed between alcohol consumption and combined diabetes and IFG among Chinese men, while alcohol intake in women was associated with a reduced risk of having diabetes. Obviously, more studies are needed to clarify the role of alcohol intake and mechanism(s) involving in the pathogenesis of diabetes mellitus.

## Competing interests

The authors declare that they have no competing interests.

## Authors' contributions

CL performed the statistical analysis and drafted the manuscript. ZY participated in the data analyses, drafting and revising the manuscript. HL, JW, LS and QB carried out the measurements of metabolic traits and cytokines. XL, as the principal investigator for the project of the Nutrition and Health of Aging Population in China, contributed to the scientific ideas, grant application, study design, result interpretation and revised the manuscript critically. All authors have read and approved the manuscript.

## Pre-publication history

The pre-publication history for this paper can be accessed here:

http://www.biomedcentral.com/1471-2458/10/713/prepub

## Supplementary Material

Additional file 1 **Multivariable adjusted odds ratios (95% confidence intervals) for diabetes mellitus and combined diabetes and IFG according to alcohol consumption categories**. This table presented the associations of alcohol consumption with the risk of having diabetes and combined diabetes and IFG, using different alcohol consumption categories as reference group. Click here for file
