# Supplementary material for: Associations of alcohol consumption with diabetes mellitus and impaired fasting glycemia among middle-aged and elderly Chinese
Source: BMC Public Health. 2010 Nov 19;10:713. doi: 10.1186/1471-2458-10-713 (PMC2998495; doi:10.1186/1471-2458-10-713)
Supplement: Additional file 1 — Multivariable adjusted odds ratios (95% confidence intervals) for diabetes mellitus and combined diabetes and IFG according to alcohol consumption categories. This table presented the associations of alcohol consumption with the risk of having diabetes and combined diabetes and IFG, using different alcohol consumption categories as reference group. [file 1471-2458-10-713-S1.DOC]

# Additional files

**Additional file 1 –Multivariable adjusted odd ratios (95% confidence intervals) for diabetes mellitus and combined diabetes and IFG according to alcohol consumption categories**

|  | Men | | | |
| --- | --- | --- | --- | --- |
|  | Abstainers | 0.1-19.9g/d | 20.0-39.9g/d | ≥40.0g/d |
| Diabetes cases/non-cases, n | 95/593 | 74/312 | 13/129 | 32/187 |
| Model 1 | 0.79(0.54,1.14) | 1.0 (reference) | 0.55 (0.29,1.07) | 1.24 (0.75,2.06) |
| Model 2 | 1.42(0.74,2.73) | 1.81 (0.94,3.51) | 1.0 (reference) | 2.53 (1.09,4.64) |
| Model 3 | 0.63 (0.38,1.04) | 0.80(0.48,1.33) | 0.44(0.22,0.91) | 1.0 (reference) |
| Combined diabetes and IFG cases/non-cases, n | 270/418 | 211/175 | 52/90 | 107/112 |
| Model 1 | 0.73 (0.55,0.98) | 1.0 (reference) | 0.59 (0.38,0.91) | 1.10 (0.75,1.61) |
| Model 2 | 1.25 (0.82,1.91) | 1.71 (1.10,2.64) | 1.0 (reference) | 1.87 (1.16,3.02) |
| Model 3 | 0.67 (0.46,0.96) | 0.91 (0.62,1.33) | 0.53 (0.33,0.86) | 1.0 (reference) |

The values are adjusted for age, geographic location (north/south), residential region (urban/rural), educational level, smoking, physical activity, family income, family history of CVD or diabetes, diet (total energy intake without alcohol, energy adjusted dietary fiber and fat intake), BMI, inflammatory markers (log-transformed CRP, IL-6 and ferritin), and adipokines (RBP4, log-transformed adiponectin and PAI-1)

Model 1, 2 and 3 were calculated using “0.1-19.9g/d”, “20-39.9g/d” and “≥40.0g/d” group as reference group respectively.
